# Detecting transitions in protein dynamics using a recurrence quantification analysis based bootstrap method

**DOI:** 10.1186/s12859-017-1943-y

**Published:** 2017-11-28

**Authors:** Wael I. Karain

**Affiliations:** Department of Physics, Birzeit University, P.O.Box 14, Birzeit, Palestine

**Keywords:** Recurrence quantification analysis- principal component analysis - molecular dynamics

## Abstract

**Background:**

Proteins undergo conformational transitions over different time scales. These transitions are closely intertwined with the protein’s function. Numerous standard techniques such as principal component analysis are used to detect these transitions in molecular dynamics simulations. In this work, we add a new method that has the ability to detect transitions in dynamics based on the recurrences in the dynamical system. It combines bootstrapping and recurrence quantification analysis. We start from the assumption that a protein has a “baseline” recurrence structure over a given period of time. Any statistically significant deviation from this recurrence structure, as inferred from complexity measures provided by recurrence quantification analysis, is considered a transition in the dynamics of the protein.

**Results:**

We apply this technique to a 132 ns long molecular dynamics simulation of the β-Lactamase Inhibitory Protein BLIP. We are able to detect conformational transitions in the nanosecond range in the recurrence dynamics of the BLIP protein during the simulation. The results compare favorably to those extracted using the principal component analysis technique.

**Conclusions:**

The recurrence quantification analysis based bootstrap technique is able to detect transitions between different dynamics states for a protein over different time scales. It is not limited to linear dynamics regimes, and can be generalized to any time scale. It also has the potential to be used to cluster frames in molecular dynamics trajectories according to the nature of their recurrence dynamics. One shortcoming for this method is the need to have large enough time windows to insure good statistical quality for the recurrence complexity measures needed to detect the transitions.

## Background

Protein functional motions occur over a wide range of time scales, and are usually accompanied by conformational transitions in the protein [1]. Principal component analysis PCA is a standard technique used to detect conformational transitions based on molecular dynamics MD simulations [2–9]. This is done by first removing the overall rotations and translations for the protein atoms by aligning each frame in the simulation to a reference frame. The covariance matrix for a set of atoms, usually the C_α_ atoms, is then built up. It is given by

1 $$ {C}_{ij}=<\left({X}_i-{X}_{i,a}\right)\left({X}_j-{X}_{j,a}\right)> $$

where *X* are the *x, y, z* coordinates for the C_α_ atoms fluctuating about their average positions *X*_*a*_. Collective motion coordinates are prepared by diagonalizing this covariance matrix. This provides a set of eigenvalues and their corresponding eigenvectors. Each eigenvector corresponds to a collective motion direction in 3 N space, where N is the number of protein residues. The corresponding eigenvalue represents the total mean square fluctuation of all the residues in that direction. The projection of motion for the protein is then calculated along any given eigenvector to show any conformational transitions over time. A small set of eigenvectors is usually sufficient to provide the majority of the fluctuations. In its standard form, this technique detects only linear correlations. Even though it has been extended to detect nonlinear correlations, its ability in this field is still not very satisfactory [2, 6], and while it is not limited to harmonic motions, some nonlinear relationships might be misinterpreted due to the neglecting of higher order correlations [6]. In addition, PCA depends on the time window length used to compute the eigenvalues and eigenvectors [10]. Conformational transitions can also be tracked by calculating the root mean square deviation RMSD for a set of atoms, such as the backbone C_α_ atoms, from a reference structure. Large changes in the RMSD usually point to conformational transitions. However, intermediate values of RMSD are sometimes hard to interpret [11].

In this work, we propose a different approach to detecting conformational transitions. We start by assuming that during any given time period, a protein has a ‘baseline’ recurrence structure. When it is undergoing a conformational transition, it will deviate from this baseline recurrence state. If this significant deviation is detected, then one can point to a conformational transition taking place. To achieve this goal, we will use recurrence quantification analysis RQA, which has gained popularity in studying dynamics, transitions, and synchronization in dynamical systems [12–14]. RQA is a quantitative version [15] of recurrence plots RP, which visually highlight recurrences in dynamical systems [12]. It is used in many fields [14]. In particular, RPs and RQA have been used extensively to study proteins over the years [16–54].

The RP is a non-linear analysis method. It visualizes graphically the recurrence S_*i*_ to a former state S_*j*_ in the phase space trajectory of the dynamical system. It is most useful when the system is being investigated experimentally, with an unknown theoretical time evolution law. If a scalar time-series {*U*_*i*_} is available for one of the measurable quantities for this system, then its trajectory can be reconstructed [55]. This reconstruction involves using the method of time delays. In essence, the dynamics of the system are assumed to be encapsulated in the time-series for the single measurable quantity, with the time delays approximating derivatives [56]. The m-dimensional phase space orbit is re-constructed from the scalar time series *U*_*i*_, such that.

2 $$ {S}_i=\left({U}_i,{U}_{i+d},..\dots {U}_{i+\left(k-l\right)d}\right)\kern0.5em k=l,..m $$

where *d* is the delay parameter between the time-delayed versions, and *m*is the embedding dimension for the reconstructed phase space. The embedding dimension *m* represents the degrees of freedom (or the number of dominant operating variables) in the dynamical system of interest. It is estimated by the method of false nearest neighbors [57]. The delay parameter *d* determines the number of points to be skipped in the scalar time-series *U*_*i*_ between the numbers forming the m-dimensional vector S. It is set to a value that makes the correlation between the points of the measured time-series at a minimum, and is estimated by finding the first minimum in the mutual information function [58]. This m-dimensional vector in phase space, *S*_*i*_, represents the state of the system at time *i*. The RP is prepared by assigning a dot at each point (*i*, *j*) whenever a point S_*j*_ lies within a ball of radius *ε* centered at *S*_*i*_. In other words, if two vectors representing the state of the system are within a certain tolerance from each other, then this means that the system is in similar states at two different time instances, *i* and *j*. The mathematical expression of the RP matrix is:

3 $$ {R}_{i,j}\left(\varepsilon \right)=\Theta \left(\varepsilon -\left\Vert {S}_i-{S}_i\right\Vert \right)\kern1em i,j=1,.\dots \dots \dots, N $$

where *N* is the number of states, *ε* is a threshold distance, Θ is the Heaviside function (Θ(x) = 0 if *x* < 0 and 1 otherwise), and ∥.∥ is chosen from one of the frequently used norms: the L_1_-norm(Minimum norm), the L_2_ norm (Euclidean norm), and the L_∞_ (Maximum norm). The norm parameter determines the size and shape of the neighborhood surrounding each reference point. In this work the maximum norm is used. The ratio of the number of dots to the total number of points in the matrix gives the recurrence rate value *RR*. The threshold or radius parameter ε is the limit that transforms the distance matrix (DM) between the time points into a recurrence matrix (RM). It plays a role similar to that of the Heaviside function. Elements (i, j) in the DM with distances between states at or below the radius cutoff are included in the RM (R_i,j_ = 1). Elements above the cutoff are excluded from RM (R_i,j_ = 0). This threshold can be chosen using a number of different techniques. For example, one rule of thumb is to choose a threshold that gives a RR value of 1% [14]. However, the value of ε is usually chosen according to the application at hand [14].

In addition to *RR*, RQA provides other output parameters. We will concentrate on two of these: determinism, DET, and laminarity, LAM. DET is the fraction of recurrence points forming diagonal lines parallel to the central diagonal. It is given by

4 $$ DET={\sum}_{l=l\min}^N lP(l)/{\sum}_{l=1}^N lP(l) $$

where *l*_*min*_ defines the minimal length for a diagonal line and is usually taken to be 2 [14]. *P(l)* is the probability distribution for the diagonal line lengths. The length of diagonal lines depends on the dynamics of the system [14]. A large number of long diagonal lines points to a high predictability of the system, and to the fact that it evolves at a similar fashion at different points in time. While the value of DET might point to a deterministic nature of a system, this is not a sufficient condition [59].

LAM is the fraction of recurrence points forming vertical structures, and is given by

5 $$ LAM={\sum}_{l=l\min}^N vP(v)/{\sum}_{l=1}^N vP(v) $$

where *l*_*min*_ defines the minimal length for a vertical line and is usually taken to be 2 [14]. *P(v)* is the probability distribution for the vertical line lengths. Vertical structures in the recurrence plot point to slowly changing states, common during laminar phases [14].

Changes in complexity measures provided by RQA, such as DET and LAM, are generally interpreted as pointing to transitions in the dynamics. In some cases, the relative values of *RR* and DET are used to detect transitions in the dynamics [60]. However, this has usually been done without providing confidence intervals to validate the significance of these changes. Recently, a method based on bootstrapping has been proposed to remedy this deficiency [61, 62]. It easily provides confidence intervals for analysis within a single dynamical system.

The method starts by preparing the recurrence matrix over a moving time window of length *w,* with the starting point of the time window at the beginning of the time series. This starting point is then shifted by a suitable number of time steps forward, and the process repeated, until the end of the time series is reached. For each time window, the local distribution for the diagonal line lengths is prepared. The distributions from all the windows are then merged together to prepare one global distribution of diagonal line lengths. This distribution is consequently used to calculate the global complexity measure of interest for the system, which will be DET in our case. From this global distribution, a large number of bootstrap distributions are drawn, and the value for DET is calculated for each draw. The α quantiles are subsequently calculated, and their corresponding confidence levels prepared for the DET distribution [62]. It is assumed that DET values above the high confidence level, and those below the low confidence level, point to a significant change in the dynamics of the system from its assumed baseline recurrence dynamics state. A similar procedure is applied to the vertical line distributions to prepare confidence levels for LAM [62].

**Fig. 1:**
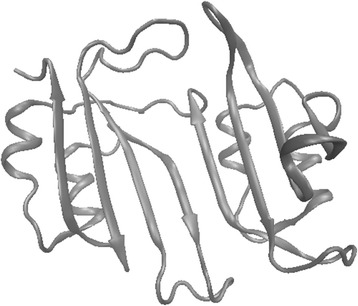
Schematic figure for BLIP protein. The figure shows the secondary structure elements for the BLIP protein. It consists of five alpha-helices, and eight beta-sheets. The connecting loops are also shown in the figure. The figure is prepared using VMD [65]

In this work we will apply this RQA based bootstrap approach to detect changes in dynamics over a 132 ns long molecular dynamics simulation, for the 165 residue β-Lactamase Inhibitory Protein BLIP at 310 K. This protein is secreted by the soil bacterium *Streptomyces clavuligerus.* It inhibits β-lactam enzymes, which hydrolyze β-lactam antibiotics and nullify their effect [63–65]. It consists of five alpha-helices, and eight beta-sheets. It also has distinct connecting loops (Fig. 1).

## Methods and calculations

**Fig. 2:**
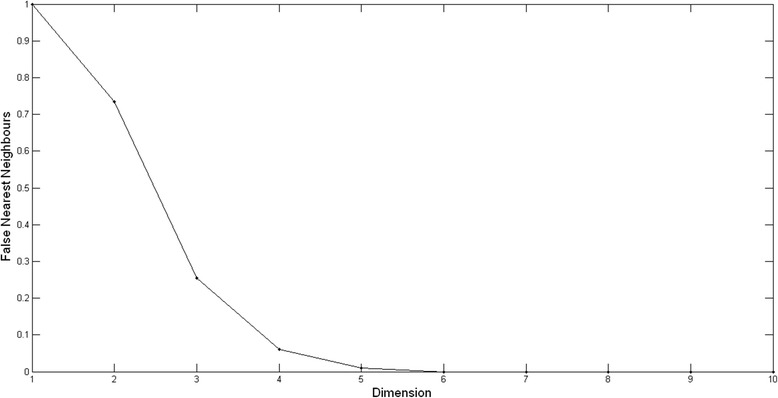
The embedding dimension. The embedding dimension value of 6 is calculated using the false nearest neighbor method in the cross recurrence CRP toolbox [69]

The computer programs VMD [66], and NAMD [67], are used to perform the molecular dynamics simulation, and the associated analyses respectively. The CHARMM27 par_all27_prot_lipid.inp parameter file is used for the force field. The starting BLIP protein structure is downloaded from the protein data bank (PDB entry 3gmu) [68]. Periodic boundary conditions are used in an 80 Å × 80 Å × 80 Å box. The protein is neutralized using 20 Cl^−^ ions and 22 Na^+^ ions. The protein is solvated using 15,264 TIP3P waters (0.15 M/ NaCl). The Particle-Mesh-Ewald method is used to do the electrostatic calculations [69]. A switching function is used for non-bonded interactions with a switch distance of 10 Å and a cutoff distance of 12 Å. A pair-list distance of 14 Å is used. The simulation is performed at constant pressure of 1 atm with an integration step of 2 fs. Langevin dynamics are used to control both temperature and pressure. A langevin temperature damping coefficient of 10/ps is applied. A langevin piston period of 200 fs and a langevin piston decay period of 100 fs, are used respectively. The protein is minimized using the conjugate gradient method for 5000 steps (10 ps) to relax any high energy areas in the system. This is followed by a gradual heating protocol in small temperature steps of 10 K to avoid thermal instability. Starting from an initial temperature of 100 K, langevin dynamics is used to increase the temperature by 10 K steps, and to control the temperature using a damping coefficient of 10/ps. The simulation is run for 10 ps for each 10 K temperature step. This is continued until reaching the final simulation temperature of 310 K. The equilibration period is 5 ns long. To insure equilibration, a number of parameters (potential energy, kinetic energy, temperature, pressure, RMSD) are tested for convergence. A 132 ns production run is consequently prepared with an integration time step of 2 fs. A root mean square deviation RMSD series is prepared using VMD for the carbon alpha atoms in the protein. The time series is 13,200 points long, with a time spacing of 10 ps between time points, for a total time length of 132 ns. The parameters for the phase space trajectory reconstruction are prepared using the CRP toolbox subroutines [70]. The maximum norm is used. The embedding dimension is prepared using the false nearest neighbor FNN subroutine and has a value of 6 (Fig. 2).

**Fig. 3:**
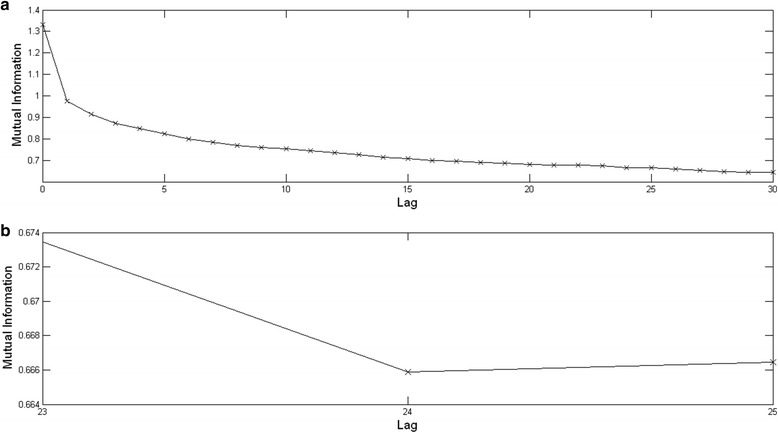
The delay parameter. The delay parameter value of 24 is calculated using the mutual information method in the cross recurrence CRP toolbox [69]. **a** This top graph shows the mutual information values for the lag range 0 to 30. **b** This bottom graph shows a magnified version of the top graph in (**a**), exhibiting the first mutual information minimum at a lag of 24. This value is used for the delay parameter

The delay parameter is prepared using the mutual information MI subroutine, and has a value of 24(Fig. 3).

The epsilon value for each time window is adaptively chosen to give a constant RR of 5%. The data inside each window is normalized. The DET and LAM parameters are subsequently calculated for a time window that is 1000 points long (10 ns) using the CRQA subroutine in the CRP toolbox [70]. The time window is then shifted by 1 ns, and the DET/LAM calculation repeated using the same procedure above until we reach the end of the time series. The diagonal line lengths and the vertical line lengths, from all the time windows, are then binned into their corresponding one global distribution. Each global distribution is then bootstrapped 1000 times. For each bootstrap copy, the number of recurrence structures drawn is the mean number of recurrence structures contained in the local distributions, and subsequently, DET and LAM are calculated [62]. Once the DET and LAM distributions are prepared, the 95% quantiles are calculated, and the corresponding upper and lower confidence levels are derived for both DET and LAM respectively. The PCA analysis is performed using the CARMA program [71].

## Results and discussion

**Fig. 4:**
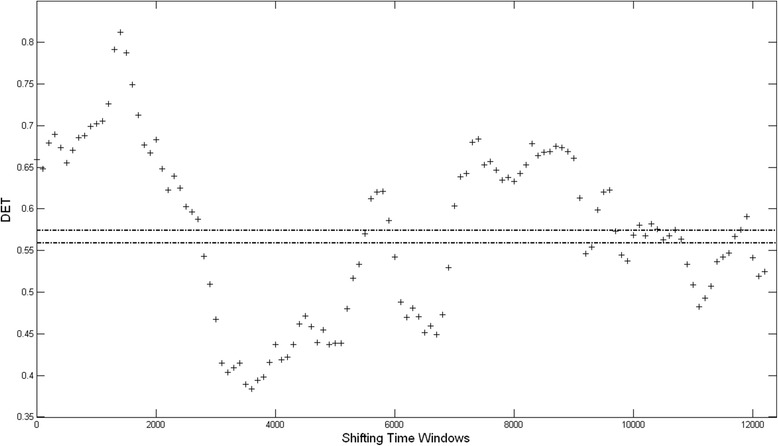
The DET parameter versus time. For each data point, the vertical coordinate gives the value for DET calculated over a time window starting at the horizontal time coordinate for the data point, and extending 10 ns into the “future”. The two horizontal dash-dot lines at 0.574 and 0.56 give the 95% upper and lower confidence levels, respectively

Figure 4 shows the results for the DET recurrence parameter versus time over the 132 ns simulation. The data points are spaced 1 ns apart. Each data point gives the value of DET for a time window that begins at that time instant, and extends 10 ns into the “future”. For example, the value for DET at 10 ns represents the DET value for the time window starting at 10 ns and ending at 20 ns. The starting point for each window is shifted forward by 1 ns relative to the previous window. Thus the DET value at 11 ns is for the window starting at 11 ns and extending to 21 ns. The two dash-dot horizontal lines at 0.574 and 0.56 show the upper and lower 95% confidence levels respectively. The time points where the value of DET is above 0.574 or below 0.56, delineate regions with significantly different recurrence dynamics than the assumed baseline recurrence dynamic state. In other words, a transition in dynamics occurs where the DET value crosses these two horizontal dash-dot lines. Both upper and lower confidence levels are given since the exact nature of the dynamics is not known [62]. Inspection of Fig. 4 shows three time regions with a DET value larger than the upper confidence level: 0 ns–27 ns, 55 ns–60 ns, and 72 ns–91 ns. In addition, there are three regions with DET values below the lower confidence level: 28 ns–55 ns, 60 ns–69 ns, and 108 ns–115 ns. Again, one needs to emphasize here that each time point in these six regions actually denotes a time window starting at that point, and extending 10 ns into the ‘future’. We recalculate DET with a constant *RR* of 1% within each time window. This is done to insure that the results are independent of the relatively high *RR* 5% value chosen for this application. The calculated DET values at 1% are shifted downwards relative to those at 5%. The 95% confidence levels are also shifted downwards. However, the time regions above and below the corresponding confidence intervals are essentially the same for both 5% and 1%. Thus the choice of 5% is justified since it has the added advantage of improving the statistical reliability of the calculations by increasing the number of recurrence structures in each time window. We also repeat the bootstrap analysis with a constant recurrence threshold value in each time window, instead of a constant *RR* value. This results in large fluctuations in the number of recurrence structures -diagonal and vertical lines- within each time window, and strongly limits the use of this bootstrap technique, which depends on having a constant statistical sample within each time window.

In these six regions, the dynamics of the protein is significantly different than the “baseline” state which is assumed to fall between the two confidence level lines. The regions with a DET value larger than the upper limit hint at an increased regularity and auto-correlation in the system, while those below the lower limit point to more irregularity and stochastic variability in the system dynamics [62]. One needs to point out that while this method detects transitions in dynamics, it does not provide a clear picture for the nature of the dynamics; only that the system has deviated from its baseline recurrence state. Another point to keep in mind is that the exact time point where the transition takes place is not well defined because each DET value is calculated over long overlapping time windows.

**Fig. 5:**
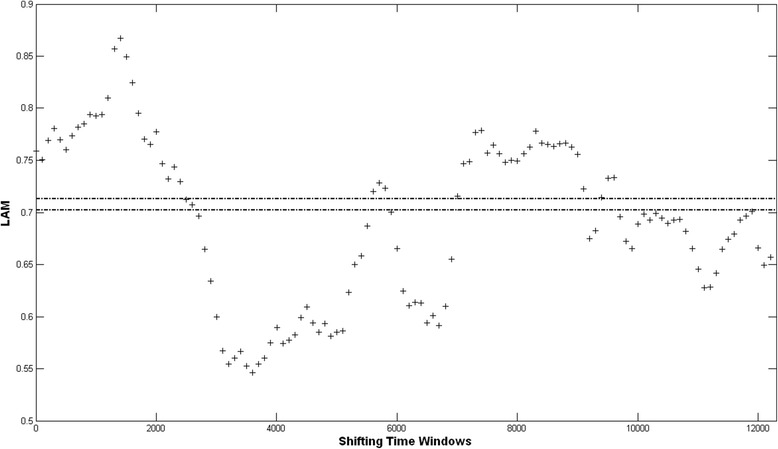
The LAM parameter versus time. For each data point, the vertical coordinate gives the value for LAM calculated over a time window starting at the horizontal time coordinate for the data point, and extending 10 ns into the “future”. The two horizontal dash-dot lines at 0.713 and 0.702 give the 95% upper and lower confidence levels, respectively

Figure 5 shows the results from the analysis on LAM. It is clear that both DET and LAM give very similar outcomes in terms of the time windows with LAM values above and below the 95% confidence levels respectively. We will therefore only use the results we get from DET for the rest of the paper.

**Fig. 6:**
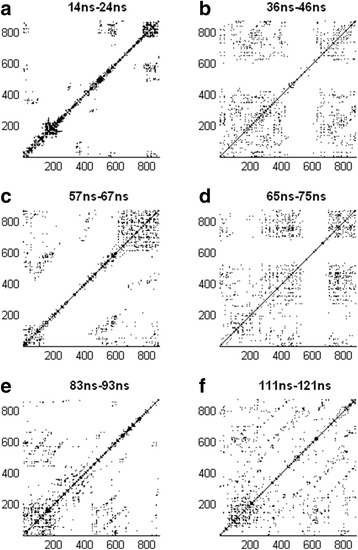
Recurrence plots. Each recurrence plots represents a 1000 points long time window(10 ns). The six time windows chosen are the ones that have the maximum and minimum DET values in the six time regions with DET values larger or smaller than the confidence levels respectively. **a** 14 ns–24 ns, (**b**)36 ns–46 ns, (**c**) 57 ns–67 ns, (**d**) 65 ns–75 ns, (**e**) 83 ns–93 ns, and (**f**) 111 ns–121 ns

Representative recurrence plots from the six regions are shown in Fig. 6. Each recurrence plot represents a time window 1000 points long(10 ns). The plots in (a), (c), and (e) are each extracted from one of the three regions with DET values above the 95% confidence level, and have the maximum DET values within their corresponding regions. The plots in (b), (d), and (f) are each extracted from one of the regions with DET values below the 95% confidence level, and have the minimum DET values within their corresponding regions. While it is difficult to draw clear and objective conclusions based only on visual inspection of these recurrence plots, it can be seen that the plots from the three regions with large DET values have a large proportion of their recurrence structures near the main diagonal. On the other hand, the plots with the small DET values are spread out over the entire plot.

**Fig. 7:**
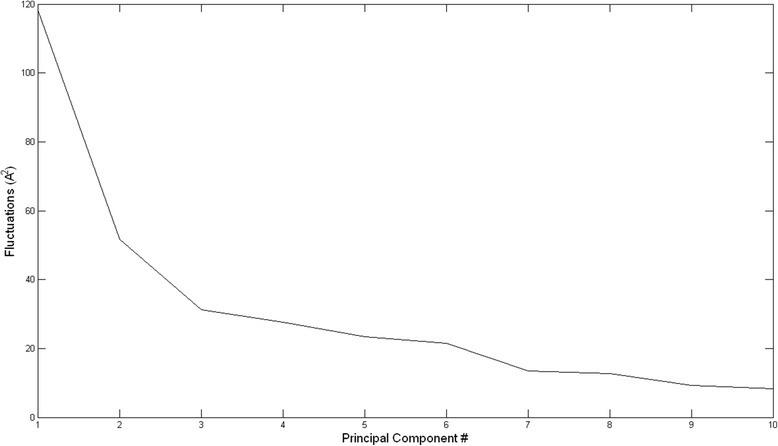
Positional fluctuations for principal component modes. The first three principal components constitute 46% of the total positional fluctuations

To gain a better picture of where these six regions lie in the conformational space, we resort to PCA. We limit our analysis to the first three principal components PCs, which constitute 46% of the total fluctuations in our simulation(Fig. 7).

**Fig. 8:**
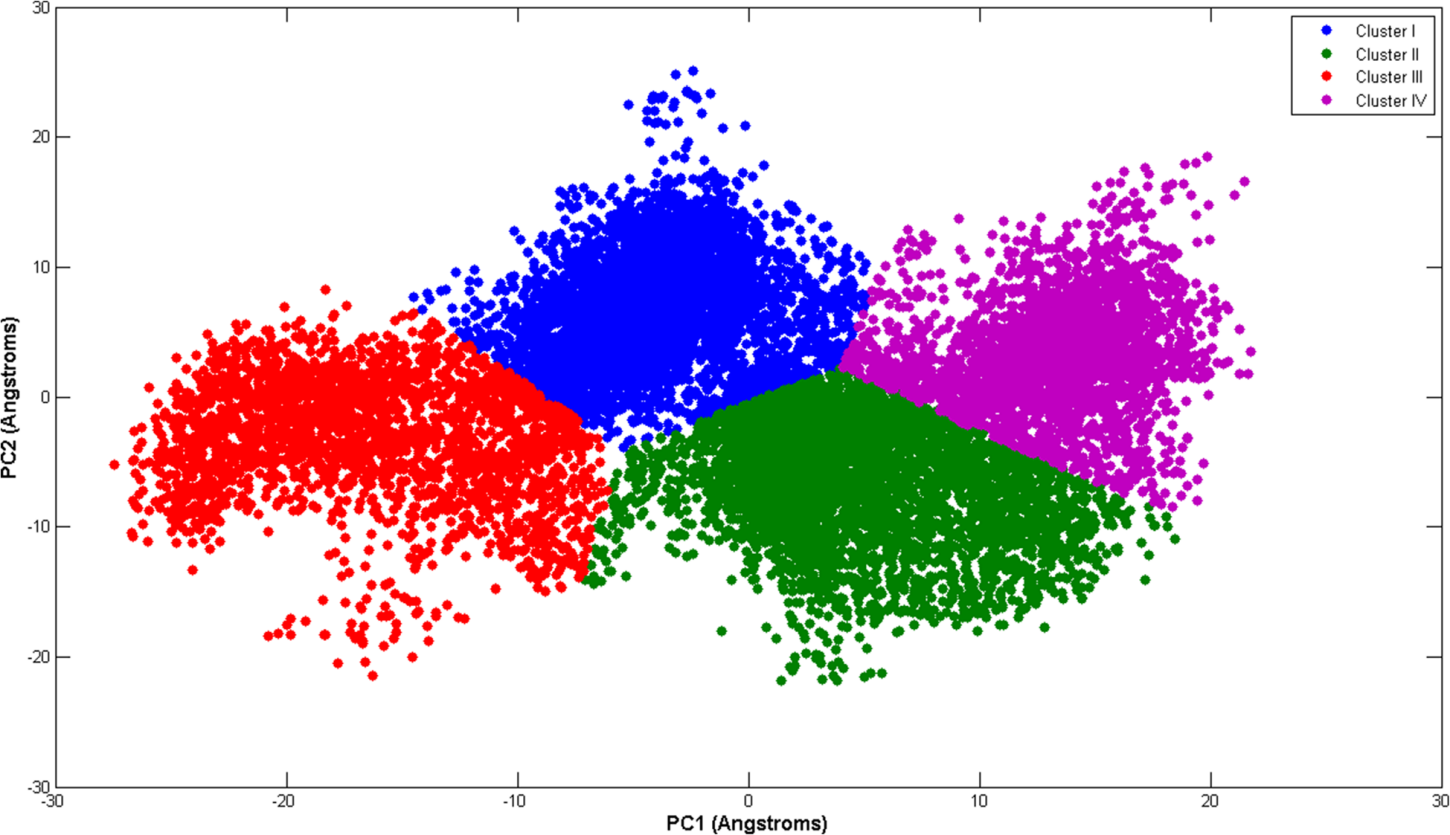
gives the two dimensional projection of the 132 ns simulation on principal component 1 PC1 and principal comonent 2 PC2, as the horizontal axis and vertical axis respectively. K-means clustering and the ‘elbow’ technique [72] are used to cluster the data. Four distinct regions emerge: cluster I, II, III, and IV, respectively

Figure 8 gives the two dimensional projection of the 132 ns simulation on principal component 1 PC1 and principal comonent 2 PC2, as the horizontal axis and vertical axis respectively. K-means clustering and the ‘elbow’ technique [72] are used to cluster the data. Four distinct regions emerge: cluster I, II, III, and IV, respectively.

**Fig. 9:**
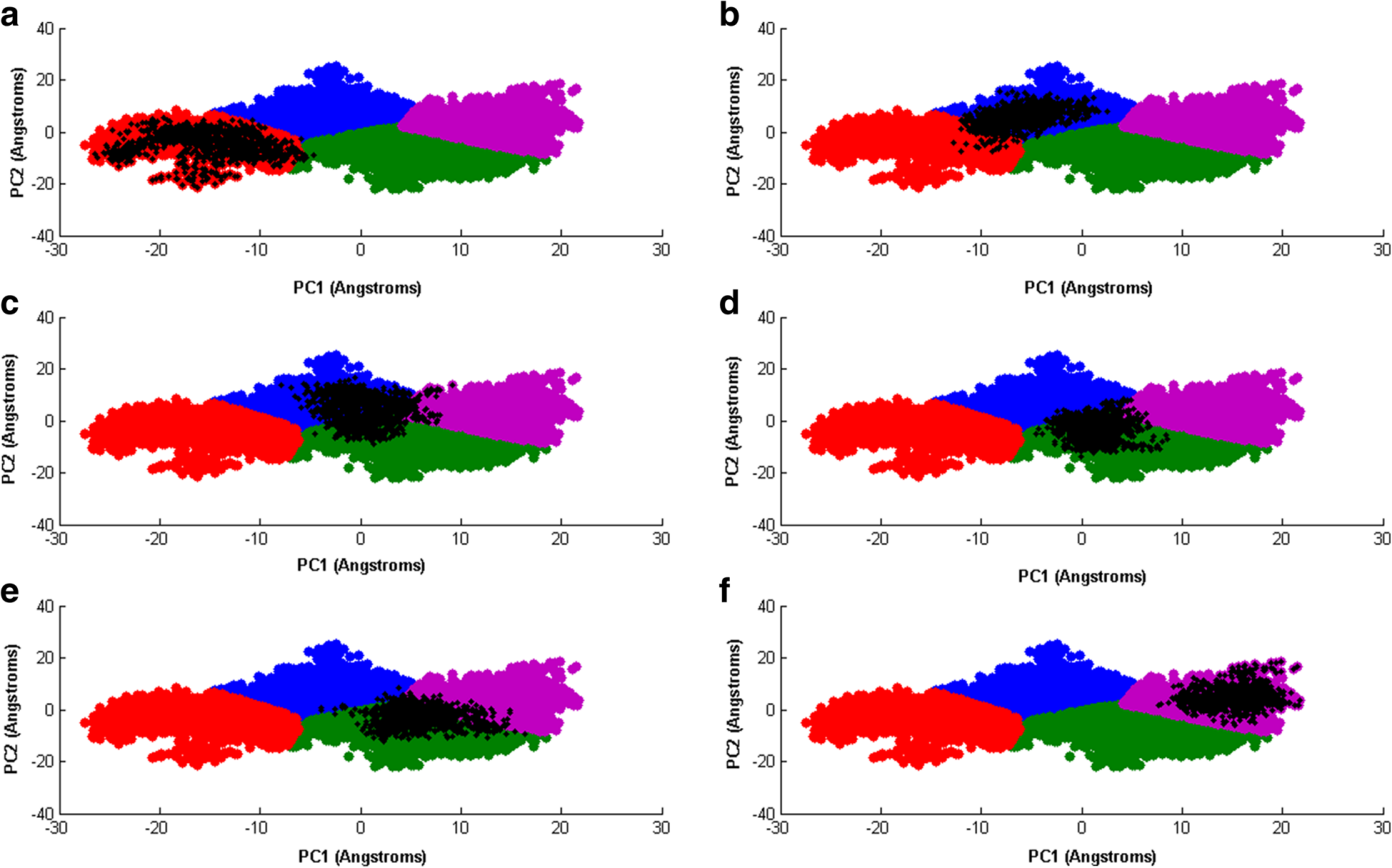
The projection of time windows over a plane defined by PC1 and PC2. The time window projections are in black: (**a**)14 ns–24 ns(large DET), (**b**) 36 ns–46 ns(small DET), (**c**) 57 ns–67 ns(large DET), (**d**) 65 ns–75 ns(small DET), (**e**)83 ns–93 ns(large DET), (**f**) 111 ns–121 ns(small DET)

In Fig. 9 we project the same six 10 ns time windows shown as recurrence plots in Fig. 5, along PC1 and PC2(projections in black color). In (a), the 14 ns–24 ns window falls inside the red cluster III. In (b), the 36 ns–46 ns window lies mainly inside the blue cluster I. In (c), the 57 ns–67 ns window straddles the blue cluster I and the green cluster II, and dips slightly into the violet cluster IV. In (d), the 65 ns–75 ns window falls mainly inside the green cluster II. In (e), the 83 ns–93 ns window also falls mainly within the green cluster II. Finally in (f), the 111 ns–121 ns window is situated inside the violet cluster IV. We notice that with the exception of the 57 ns–67 ns window, the windows fall mainly within a single cluster each. It is also interesting to note that time windows with large and small DET values fall within the same cluster(Fig. 9d and e). This shows that while PCA lumps regions with distinct dynamics within the same cluster, while the RQA-bootstrap method is able to resolve them apart.

**Fig. 10:**
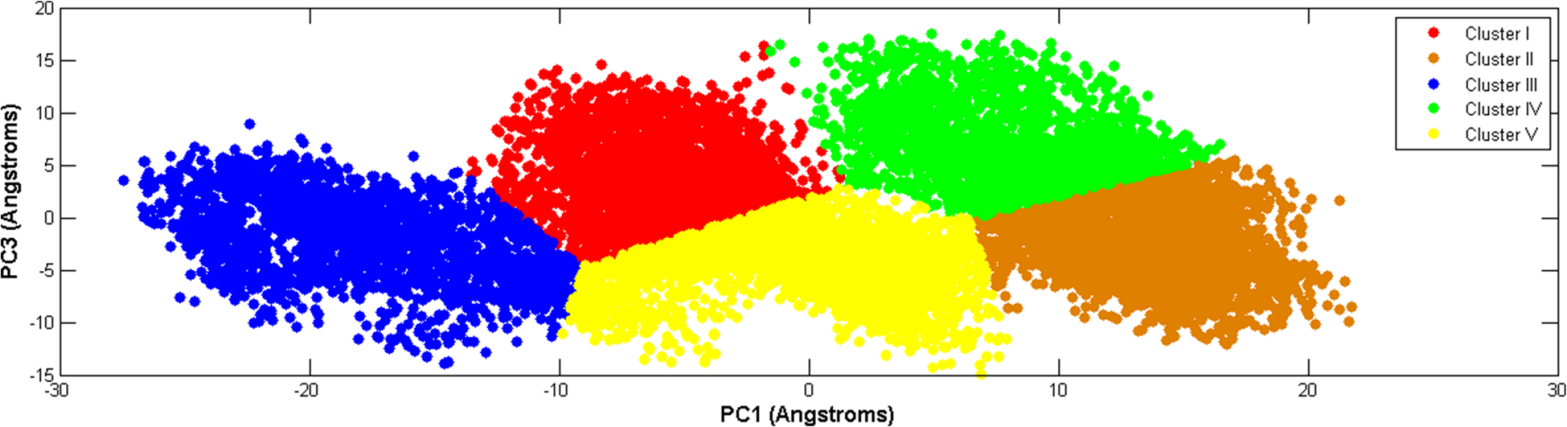
The projection of the 132 ns simulation over PC1 and PC3. The five clusters I, II, III, IV, and V are grouped using k-means clustering and the ‘elbow’ technique

Figure 10 gives the two dimensional projection of the 132 ns simulation on principal component 1 PC1 and principal comonent 3 PC3, as the horizontal axis and vertical axis respectively. K-means clustering and the ‘elbow’ technique [72] are used to cluster the data points. Five distinct regions emerge: cluster I, II, III, IV, and V, respectively.

**Fig. 11:**
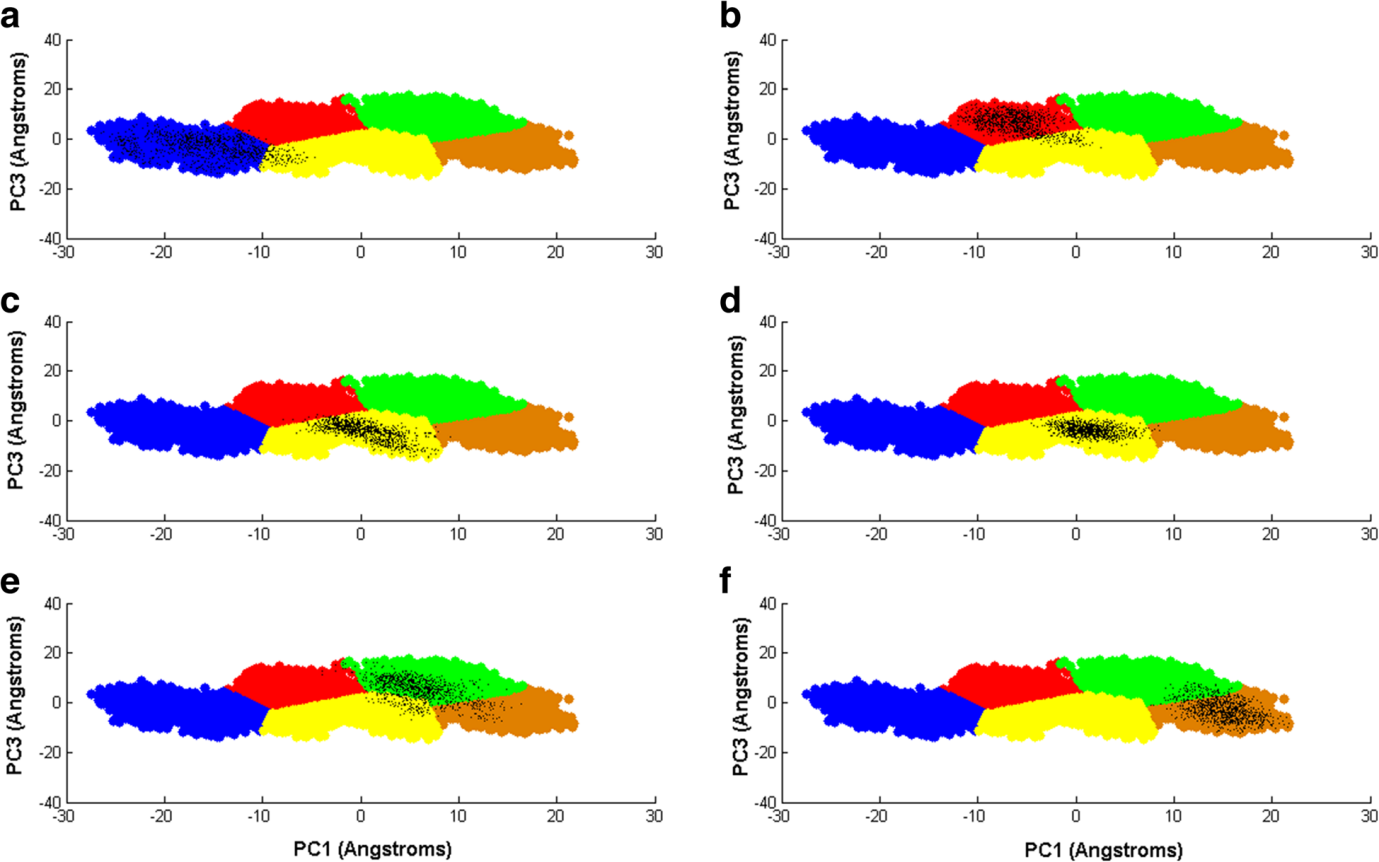
The projection of time windows over a plane defined by PC1 and PC3. The time window projections are in black: (**a**)14 ns–24 ns(large DET), (**b**) 36 ns–46 ns(small DET), (**c**) 57 ns–67 ns(large DET), (**d**) 65 ns–75 ns(small DET), (**e**)83 ns–93 ns(large DET), (**f**) 111 ns–121 ns(small DET)

In Fig. 11 we project the same six 10 ns time windows shown as recurrence plots in Fig. 6, along PC1 and PC3(projections in black color). In (a), the 14 ns–24 ns time window falls mainly inside the blue cluster III. In (b), the 36 ns–46 ns time window lies mainly inside the red cluster I. In (c), the 57 ns–67 ns time window is inside the yellow cluster V. In (d), the 65 ns–75 ns time window also falls inside the yellow cluster V. In (e), the 83 ns–93 ns time window also falls mainly within the green cluster IV. Finally in (f), the 111 ns–121 ns window is situated inside the brown cluster II. Again, we notice that the 57 ns–67 ns(large DET) and 65 ns–75 ns(small DET) time windows fall within the yellow cluster V, while the other four time windows fall mainly within a single cluster each. This again shows that while PCA lumps regions with distinct dynamics within the same cluster, the RQA-bootstrap method is able to resolve them apart.

**Fig. 12:**
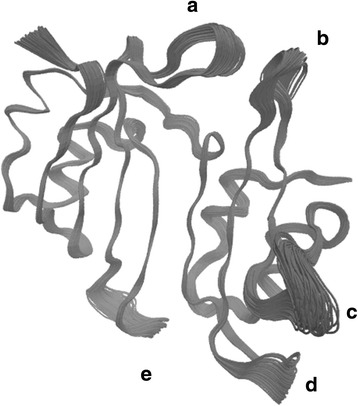
Collective protein carbon alpha motions projected over PC1, PC2, and PC3 simultaneously. The projection of time window 14 ns–24 ns, with DET value larger than the upper 95% confidence level, simultaneously along the principal component eigenvectors with the three largest eigenvalues. Regions **a**, **b**, **c**, **d**, and **e** show clear collective loop domain motions. The figure is prepared using VMD [65]

**Fig. 13:**
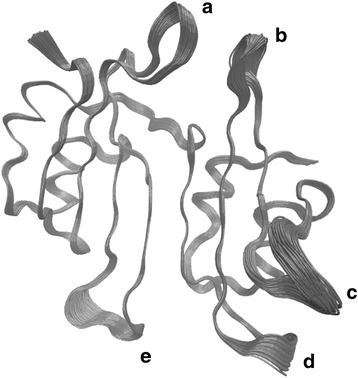
Collective protein carbon alpha motions projected over PC1, PC2, and PC3 simultaneously. The projection of time window 36 ns–46 ns with DET value smaller than the lower 95% confidence level, simultaneously along the 3 PC eigenvectors with the three largest eigenvalues. Regions **a**, **b**, **c**, **d**, and **e** show clear collective loop domain motions. The figure is prepared using VMD [65]

To gain some insight into the conformational structural nature of these transitions, we project two 10 ns long time windows over the principal components with the three largest eigenvalues, and show where the collective domain motion amplitudes are the largest. The first time window is from 14 ns to 24 ns (Fig. 12), and has a DET value larger than the upper 95% confidence level. The second time window is from 36 ns to 46 ns (Fig. 13), and has a DET value smaller than the lower 95% confidence level. It is clear that for both of these time windows, the collective domain motions are taking place mainly within the loop regions between the secondary structures of the protein. In Figs 12 and 13, there are five regions with clear collective loop domain motions. In region **a**, the loop lies between beta-sheets 6 and 7. In region **b**, the loop lies between beta-sheets 2 and 3. In region **c,** the loop lies between the short alpha-helix 3 and beta-sheet 2. In region **d**, the loop lies between beta-sheets 3 and 4. In region **e**, the loop lies between beta-sheets 5 and 6. Such loop structural conformations can play an important role in protein docking and active site stabilization [73–80]. For BLIP in particular, residue Asp-49 which lies within region **b** in the loop between beta-sheets 2 and 3, and residue Phe-142 which lies within region **a** in the loop between beta-sheets 6 and 7, play an important role in the inhibition behavior for the protein [81].

.

## Conclusions

We have introduced a RQA based bootstrap method to differentiate between different recurrence dynamics regions in a protein molecular dynamics simulation. The nature of the dynamics is specifically related to the recurrence characteristics of the dynamical system. The method compares well with PCA. In addition, while PCA shows that certain time regions fall within a single cluster in conformational space, they actually have different recurrence qualities. This method can thus be used in unison with PCA to clarify the degree of correlation and predictability during a certain time window. It can also be used to cluster molecular dynamics trajectory data based on recurrence properties, in an effort to remove redundant data within the same dynamics region [82]. This method, based on the recurrence properties of the protein dynamics system, can be an added tool in the search for understanding of the relation between dynamics and function for a protein.

## Abbreviations

CRP: Cross recurrence plot
CRQA: Cross recurrence quantification analysis
DET: Determinism
DM: Distance matrix
FNN: False nearest neighbor
MD: Molecular dynamics
MI: Mutual information
NAMD: Not (just) Another Molecular Dynamics program
PCA: Principal component analysis
PDB: Protein data bank
RM: Recurrence matrix
RMSD: Root mean square deviation
RP: Recurrence plot
RQA: Recurrence quantification analysis
RR: Recurrence rate
VMD: Visual molecular dynamics
